# A Plastid-Localized Pentatricopeptide Repeat Protein is Required for Both Pollen Development and Plant Growth in Rice

**DOI:** 10.1038/s41598-017-10727-x

**Published:** 2017-09-13

**Authors:** Yu-Jun Liu, Xuejiao Liu, Hao Chen, Peng Zheng, Wenyi Wang, Liangchao Wang, Jianhua Zhang, Jumin Tu

**Affiliations:** 1 Institute of Crop Science, College of Agriculture and Biotechnology, Zhejiang University, Hangzhou, 310058 P. R. China; 2 State Key Lab of Agrobiotechnology, School of Life Science, The Chinese University of Hong Kong, N.T. Hong Kong, P. R. China

## Abstract

Several mitochondrial-targeted pentatricopeptide repeat (PPR) proteins involved in pollen development have been reported to be fertility restorer (Rf) proteins. However, the roles of plastid-localized PPR proteins in plant male reproduction are poorly defined. Here, we described a plastid-localized PPR-SMR protein, OsPPR676, which is required for plant growth and pollen development in rice. In this study, OsPPR676 was confirmed to be an interacted protein with Osj10gBTF3, β-subunit of nascent polypeptide-associated complex (β-NAC), by bimolecular fluorescence complementation assays, indicating that both proteins are probably involved in the same regulatory pathway of pollen development. Compared with other chloroplast-rich tissues, OsPPR676 was only weakly expressed in anther, but in the Mei and YM stages of pollen development, its expression was relatively strong in the tapetum. Disruption of OsPPR676 resulted in growth retardation of plants and partial sterility of pollens. Phenotypic analysis of different *osppr676* mutant lines implied that the SMR domain was not essential for the function of OsPPR676. We further demonstrated that OsPPR676 is essential for production of plastid *atpB* subunit, and then plays crucial roles in biosynthesis of fatty acids, carbohydrates, and other organic matters via affecting activity of ATP synthase.

## Introduction

Rice is not only an important monocot model for biological studies, but also a crop of global economic and agronomic significance. It is estimated that the world rice production of 2015 was approximately 490.1 million metric tons (FAOstat, 2016; http://faostat.fao.org/), and this forms the staple food for more than half of the world’s population^1^. Over the past 30 years, the importance of hybrid rice has clearly been demonstrated with yield increases of approximately 20% over that of inbred rice^2^. Controlling the process of male reproduction is critical for selective breeding, the release of genetically modified (GM) pollen, and the commercial development of hybrid lines.

Rice male sterility is frequently caused by environmental effects or genetic mutation, leading to defective anther development and pollen fertility. The study of the genes involved in pollen development has principally focused on model plants *Arabidopsis* and more recently on rice^3, 4^. Cytoplasmic male sterility (CMS), a condition under which a plant is unable to produce functional pollen, is widespread among higher plants. A specific set of nuclear genes called restorer-of-fertility (Rf) genes, which primarily belong to the pentatricopeptide repeat (PPR) family, are required for the development of a functional male gametophyte in plants with S (sterile) type cytoplasm. In majority of cases, Rf-related PPR proteins can restore CMS phenotype by reducing the accumulation of CMS-associated RNAs and/or proteins. For example, RF1A and RF1B are both targeted to mitochondria and can restore rice male fertility by blocking ORF79 production via endonucleolytic cleavage (RF1A) or degradation (RF1B) of dicistronic *B-atp6/orf79* mRNA^5^. Another PPR protein RF5 interacts directly with a Gly-rich protein GRP162 to form a subunit of restorer fertility complex (RFC), binding to CMS-associated transcripts *atp6/orfH79* to restore rice fertility^6^. With the difference from RF5, RF6 works with OsHXK6 in mitochondria to stimulate the processing of transcript *atp6-orfH79*, leading to normal pollen development and fertility restoration^7^. Other *Rf* genes encoding PPR proteins include *PPR592* in petunia (*Petunia hybrida*), *Rf1k* in Kosena rapeseed, *Rfo* in radish (*Raphanus sativus*), and *PPR13* in sorghum (*Sorghum bicolor*)^8–12^.

All these Rf proteins are mitochondrial-targeted. In this study, we first report a plastid-localized PPR-SMR protein OsPPR676 required for both pollen development and plant growth in rice. As a small subgroup in the P-type PPR subfamily, PPR-SMR proteins are characterized by a PPR tract followed by a carboxy-terminal small MutS-related (SMR) domain^13^. All reported studies to date that investigated the function of PPR-SMR proteins have focused on *Arabidopsis* and maize. GUN1 is a central regulator of plastid retrograde signalling, where the developmental and functional state of the plastid controls the expression of nuclear gene-encoding plastid-localized proteins^14^. pTAC2 associates with the plastid-encoded RNA polymerase (PEP) and is required for its activity^15^, but the basis for this effect is unknown. The *svr7* mutant in *Arabidopsis* was identified during a screen for suppressors of *var2* variegation^16^. SVR7 is involved in the translational activation of the *atpB/E* and *rbcL* transcripts, resulting in a specific reduction in the accumulation of ATP synthase subunits A, B, E, and F^17^. ATP4, the maize orthologue of SVR7, is also required for the translation of the *atpB* open reading frame (ORF) and for the accumulation of specific processed transcript isoforms from the *atpF*, *psaJ* and *rpl14* loci^18^. Other two PPR-SMR proteins, PPR53 in maize, and SOT1, its orthologue in *Arabidopsis*, are required for maturation of the 23S-4.5 S rRNA dicistron and play essential roles in the biogenesis of the photosynthetic apparatus by promoting the expression of the chloroplast *ndhA* and *rrn23* genes^19, 20^.

To further characterize the PPR-SMR protein subgroup and elucidate the function of the SMR domain, we characterized OsPPR676, the rice orthologue of ATP4 and SVR7. In the previous study of a basal transcription factor 3-like gene *Osj10gBTF3* ^21^, OsPPR676 was identified as a candidate protein that interacts with Osj10gBTF3. Repression of Osj10gBTF3 caused significant plant miniaturization and pollen abortion^21^. In contrast to the mutant phenotypes of other plastid PPR-SMRs, which predominantly showed different levels of plant growth retardation and pale leaves^14–16, 18^, *osppr676* mutants showed not only growth retardation, but also pollen semisterility. The phenotype is similar with Osj10gBTF3 RNAi lines. Phenotypic analysis revealed that the SMR domain is not essential for OsPPR676 function. The results of anther RNA *in situ* hybridization indicated that *OsPPR676* transcripts are principally expressed in the tapetum, and the total soluble lipids of *osppr676* anthers were greatly reduced compared to the WT plants. Overall, this work characterized a rice PPR-SMR protein for the first time, and unravelled a probable new mechanism of male sterility in plants.

## Results

### *OsPPR676* encodes a plastid-localized PPR-SMR protein

**Figure 1:**
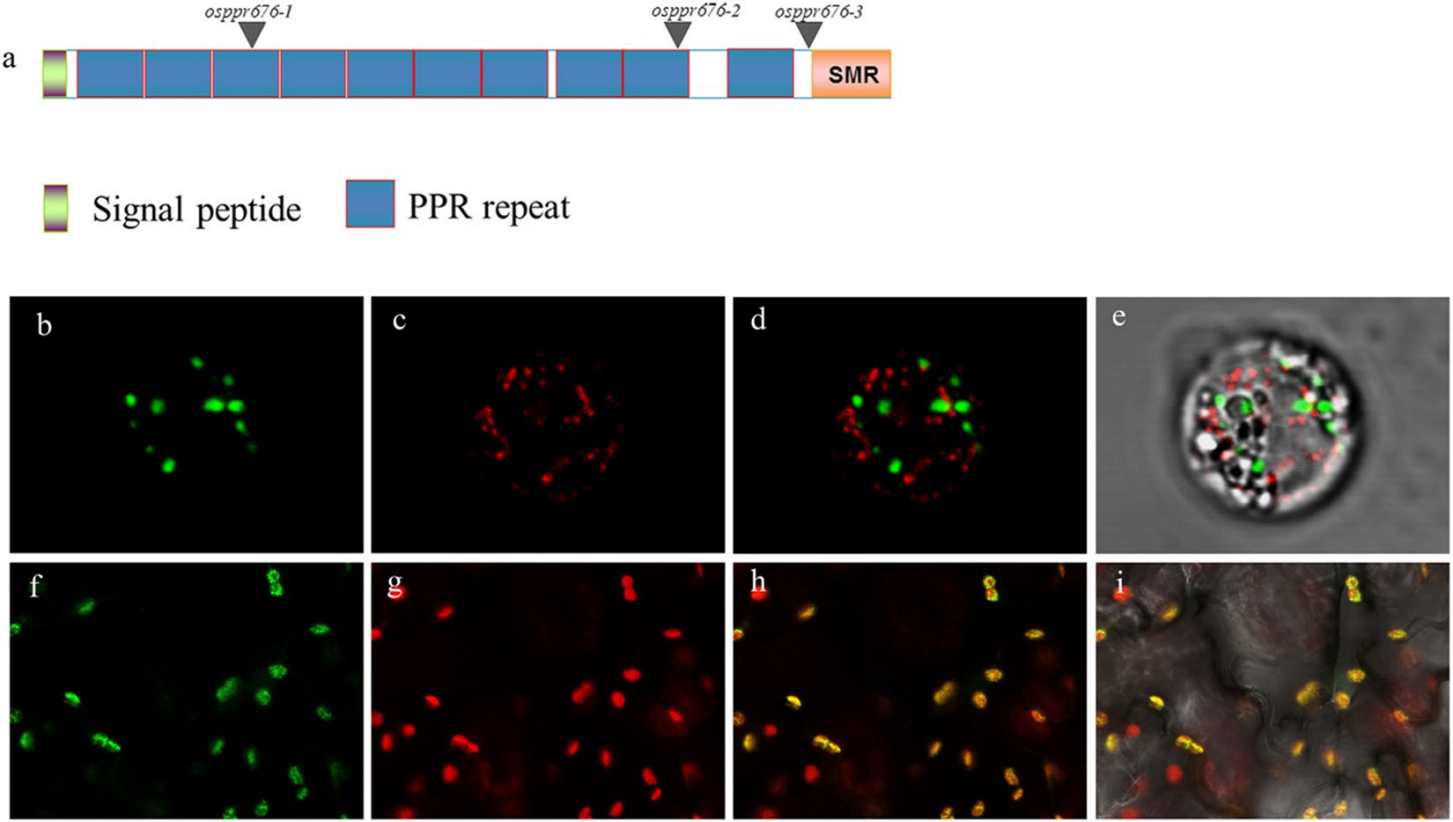
OsPPR676 is a plastid-localized PPR-SMR protein. (**a**) The OsPPR676 protein contains 10 PPR motifs and a SMR domain at the C-terminus. Locations of mutation sites for 3 studied alleles marked with triangles. (**b**) to (**e**) OsPPR676:GFP and a mitochondrial marker F1-ATPase-γ:RFP co-transient expression in rice protoplast. (**f**) to (**i**) OsPPR676: GFP transient expression in tobacco epidermis cells. The tobacco leaf samples and protoplast were used to view GFP signals respectively. Fluorescence signals from OsPPR676-GFP (green) and F1-ATPase- γ:RFP (red) were detected by confocal microscopy.

To study the function of OsPPR676,we analyzed OsPPR676 sequence, indicating that the OsPPR676 is the orthologue of maize ATP4 and *Arabidopsis* SVR7. Motif prediction by the algorithm TPRpred (http://tprpred.tuebingen.mpg.de/tprpred) showed that OsPPR676 contains 10 PPR motifs and has a C-terminal small MutS-related (SMR) domain, classifying this protein as a member of the PPR-SMR protein subgroup (Fig. 1a).

To experimentally determine the subcellular localization of OsPPR676, we fused full-length OsPPR676 with green fluorescent protein (GFP) and transiently expressed the construct in tobacco epidermis cells. Confocal laser scanning microscopy analysis of the transformed tobacco cells showed co-localization of the GFP signal of OsPPR676-GFP with the chlorophyll autofluorescence in the plastids (Fig. 1h). Previously, F1-ATPase-γ: RFP was shown to target to the mitochondria^22^. The OsPPR676-GFP construct and the mitochondrial marker F1-ATPase- γ: RFP were also transiently co-expressed in rice protoplasts, which showed that the GFP signal of OsPPR676-GFP was not localized to the mitochondria (Fig. 1d). Together, these results indicate that *OsPPR676* encodes a plastid-localized PPR-SMR protein, as the conclusion made for its maize, *Arabidopsis* and radish orthologues previously^16, 18, 23^.

### OsPPR676 interacts with Osj10gBTF3

**Figure 2:**
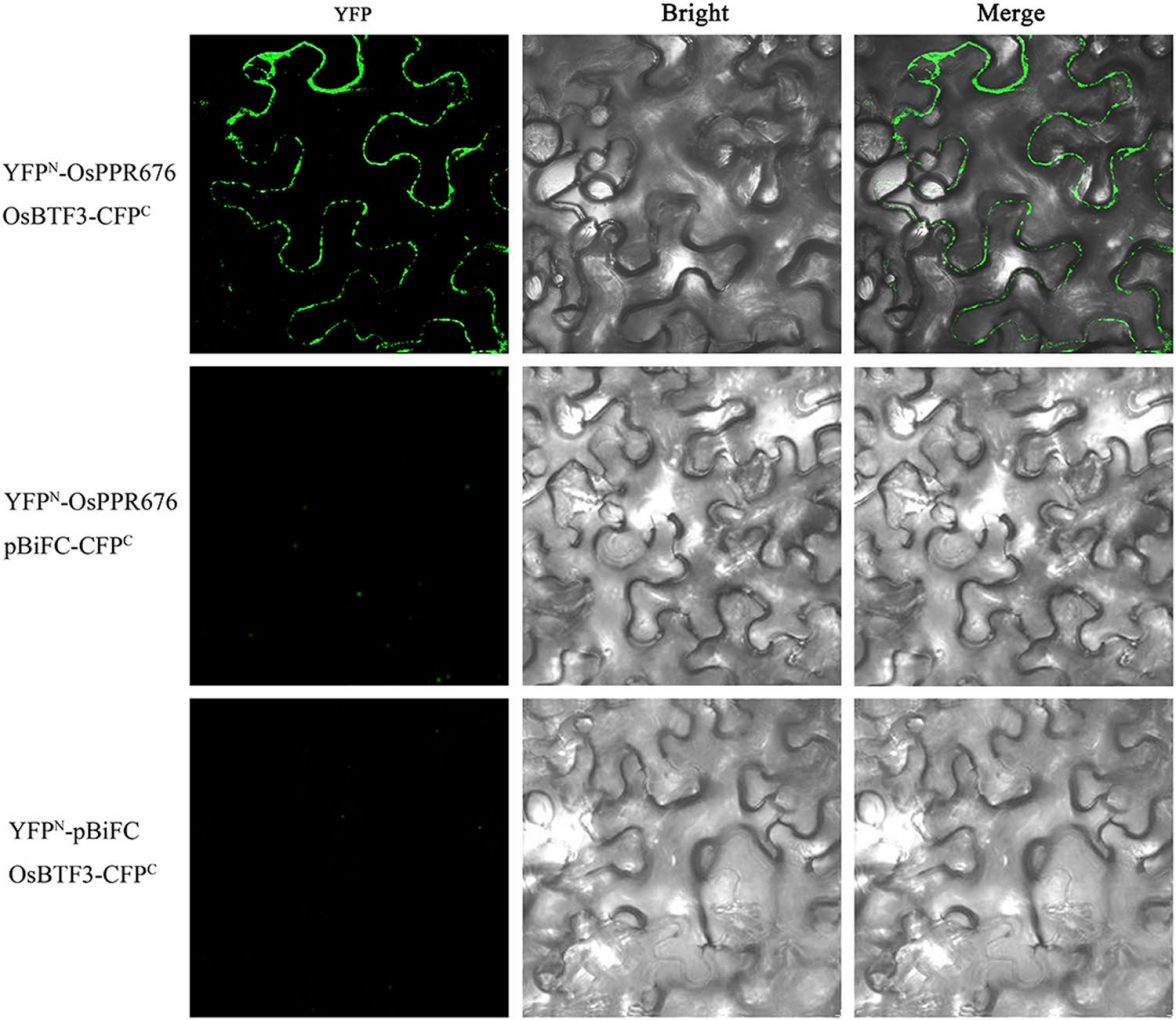
Bimolecular fluorescence complementation (BiFC) analysis of OsPPR676 and Osj10gBTF3. YFP^N^-OsPPR676, OsPPR676 fused with N-terminal of YFP; Osj10gBTF3 -CFP^C^, Osj10gBTF3 fused with C terminal of CFP. CFP^C^ is modified from C-terminal of YFP in order to enhance fluorescence signal. As controls, combinations with the YFP^N^-OsPPR676 and the empty vector pBiFC-CFP^C^ or Osj10gBTF3 -CFP^C^ and the empty vector YFP^N^-pBiFC were included.

To confirm the interaction between OsPPR676 and Osj10gBTF3, the full-length cDNAs of *OsPPR676* and *Osj10gBTF3* were cloned in binary pBiFC vectors containing the amino-terminal fragment of the eYFP fluorescent protein or carboxy-terminal fragment of the eCFP fluorescent protein (eYFP^N^ and eCFP^C^, respectively)^24^ to perform bimolecular fluorescence complementation (BiFC) assays. All constructs were transformed into *Agrobacterium tumefaciens*. Different combinations of these constructs were mixed at an OD_600_ 1:1 ratio and injected into 2–3-week-old tobacco epidermal cells. Positive cytosol BiFC interaction signals between YFP^N^-OsPPR676 and OsBTF3-CFP^C^ were detected with confocal fluorescence microscopy (Fig. 2). Meanwhile, the combinations between YFP^N^-OsPPR676 and empty pBiFC-CFP^C^ or YFP^N^- pBiFC and empty OsBTF3-CFP^C^ were used as negative controls for the BiFC assay. None of these combinations showed green fluorescence signals in tobacco epidermal cells. These results suggest that OsPPR676 and Osj10gBTF3 directly interact in the cytosol and probably involve in the same regulatory pathway of pollen development.

### Expression of *OsPPR676*

**Figure 3:**
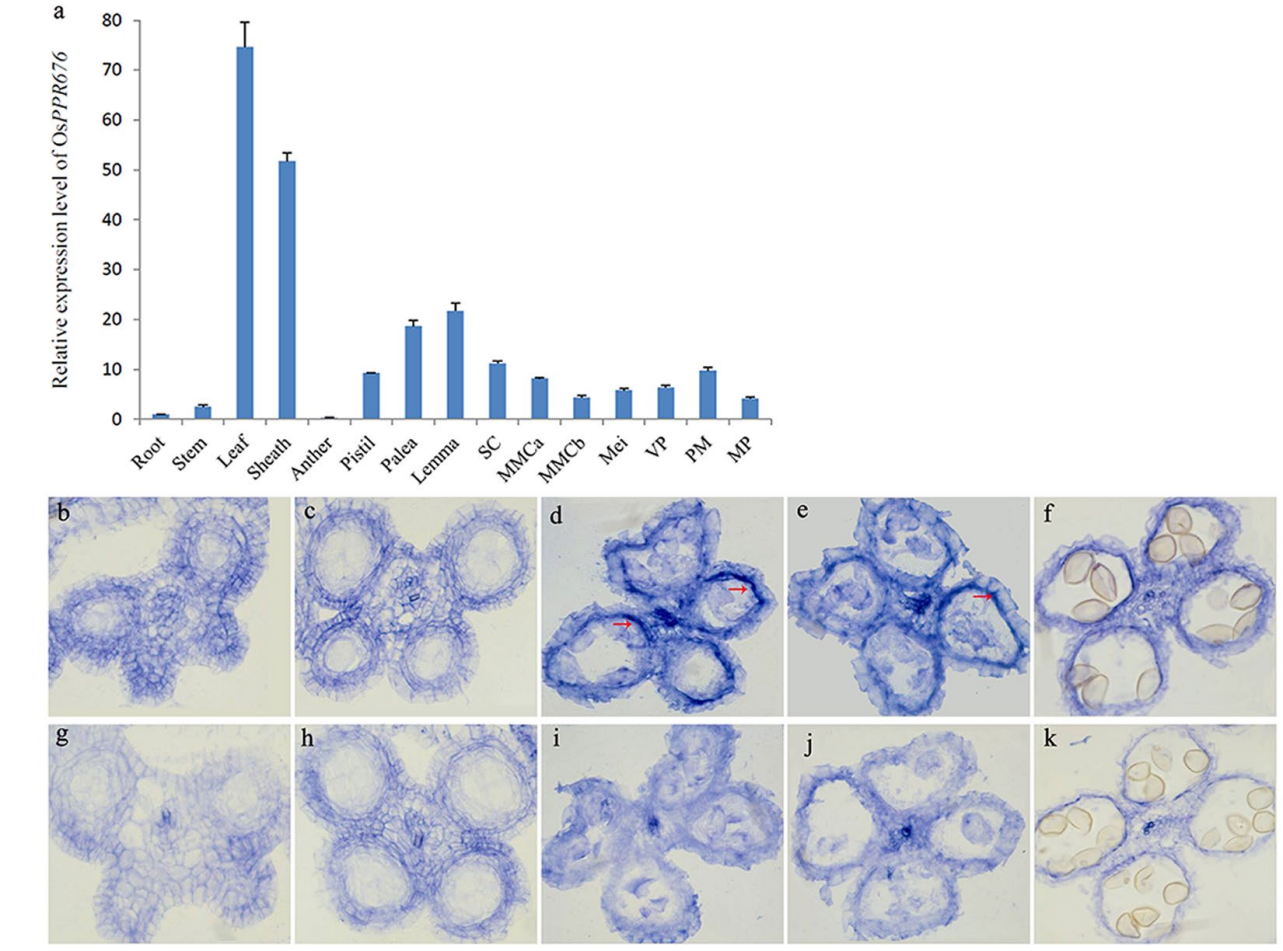
*OsPPR676* expression pattern. (**a**) Expression analysis of *OsPPR676* using qRT-PCR. RNA was extracted from spikelets of SC stage, MMC stage, Mei stage, VP stage, PM stage, MP stage, respectively. Error bars refer to SD of three biological repeats. (**b**) to (**f**) *In situ* hybridization assays of *OsPPR676* in the anthers of the WT at SC stage (**b**), MMC stage (**c**), Mei stage (**d**), YM stage (**e**), and VP stage (**f**). (**g**) to (**k**) Negative controls with sense probe in anthers at SC stage (**g**), MMC stage (**h**), Mei stage (**i**), YM stage (**j**), and VP stage (**k**). SC, sporogenous cell; MMC, microspore mother cell; Mei, meiosis; VP, vacuolated pollen; YM, young microspore; PM, pollen mitosis; MP, mature pollen.

The expression of OsPPR676 was analyzed by qRT-PCR. The results indicated that the *OsPPR676* transcripts were present in various vegetative and reproductive organs, including root, stem, leaf, sheath, anther, pistil, lemma, palea and seven different developmental stages of spikelet (Fig. 3a). Relatively high mRNA expression was observed in the leaf, sheath, lemma and palea, which all are green tissues rich in chloroplasts. Weak expression was detected in the root and anther, which are not green tissues or rich in plastids. Considering the fact that OsPPR676 is a plastid-localized protein, these results hence suggest that OsPPR676 may be expressed in all types of plastids, including the chloroplasts from green tissues and the leucoplasts and chromoplasts from non-green tissues. In addition, the OsPPR676 function may not be limited to anther development. Rather, it may also have functions in other vegetative tissues during plant growth and development.

To more precisely determine the spatial and temporal patterns of *OsPPR676* expression during anther development, we performed RNA *in situ* hybridization with WT floral sections. At the early sporogenous cell (SC) stage and the microspore mother cell (MMC) stage, no *OsPPR676* expression was detected (Fig. 3b and c). Strong expression of *OsPPR676* was detected in the tapetum cells in the meiosis (Mei) stage and young microspore (YM) stage anthers (Fig. 3d and e), but it was greatly reduced in the vacuolated pollen (VP) stage along with tapetum degradation (Fig. 3f). These results demonstrate that *OsPPR676* is indeed expressed in the tapetum, which infers that it most likely acts directly on anther development.

### *osppr676* mutants generated by CRISPR/Cas9 system on a Nipponbare background

To further study the function of OsPPR676, we generated mutants by the CRISPR/cas9 system. We designed three different target sequences, SG638, SG640 and SG641 of *OsPPR676* to construct sgRNA-CRISPR/Cas9 expression vectors. Each of these sgRNA-CRISPR/Cas9 constructs was transformed independently into rice calluses derived from mature seeds of Nipponbare. The obtained T_0_ plants were analyzed to detect mutations in the targeted sequence regions. Each target site had several similar mutant lines generated by different base mutations (Table S2). The total mutation rate for all three target sites reached 62.8% and homozygous mutants were found in the T0 plants of all three targets, accounting for approximately 20% of the plants examined (Table 1). All homozygous mutants segregated from T1 plants were shown in Table S2 (containing T0 homozygous plants), and the subsequent reliable phenotypic analysis and functional studies of protein motifs were carried out on the basis of these multiple diverse mutants.

**Table 1:** Percentage of T0 plants found with *OsPPR676* mutations in the target sequences.

Target	Guide RNA	No. of plants examined	No. of plants with mutations	Mutation rate (%)	homozygous mutations	
					Number	%
SG638	SgRNA1	21	11	52.4	3	14.3
SG640	SgRNA2	18	13	72.2	4	22.2
SG641	SgRNA3	31	20	64.5	7	22.6
total		70	44	62.8	14	20

Previous studies in mice and zebrafish indicated a low off-target effect of CRISPR/Cas9-induced mutagenesis^25, 26^. Similarly in plants, deep sequencing of CRISPR/Cas-modified whole-genome did not detect any off-targets in *Arabidopsis* and detected only one off-target mutation case in rice^27, 28^. In our study, each sgRNA-CRISPR/Cas9 construct (containing one specific target sequence) was transformed independently, resulting in multiple diverse homozygous mutants in each target site in T_1_ generation (shown in Table S2, including T_0_ homozygous). These homozygous mutants were used for phenotypic analysis and all lines mutated in the same target sequence shared the same phenotypes.

In order to detect whether CRISPR/Cas9-mediated gene editing process produced any off-target event, we analyzed possible off-target sites corresponding to each target site predicted by software CRISPR-P (http://cbi.hzau.edu.cn/crispr/) via PCR product sequencing. The results reveal that there is definitely no any off-target mutation detected in these predicted off-target sites for all examined mutant lines (Table S3) as previous report^27^. Therefore, the possibility of off-target event for the CRISPR/Cas9-induced mutants of this study did not exist.

### Phenotypic analysis of the CRISPR/Cas9-generated mutants and RNAi lines

**Figure 4:**
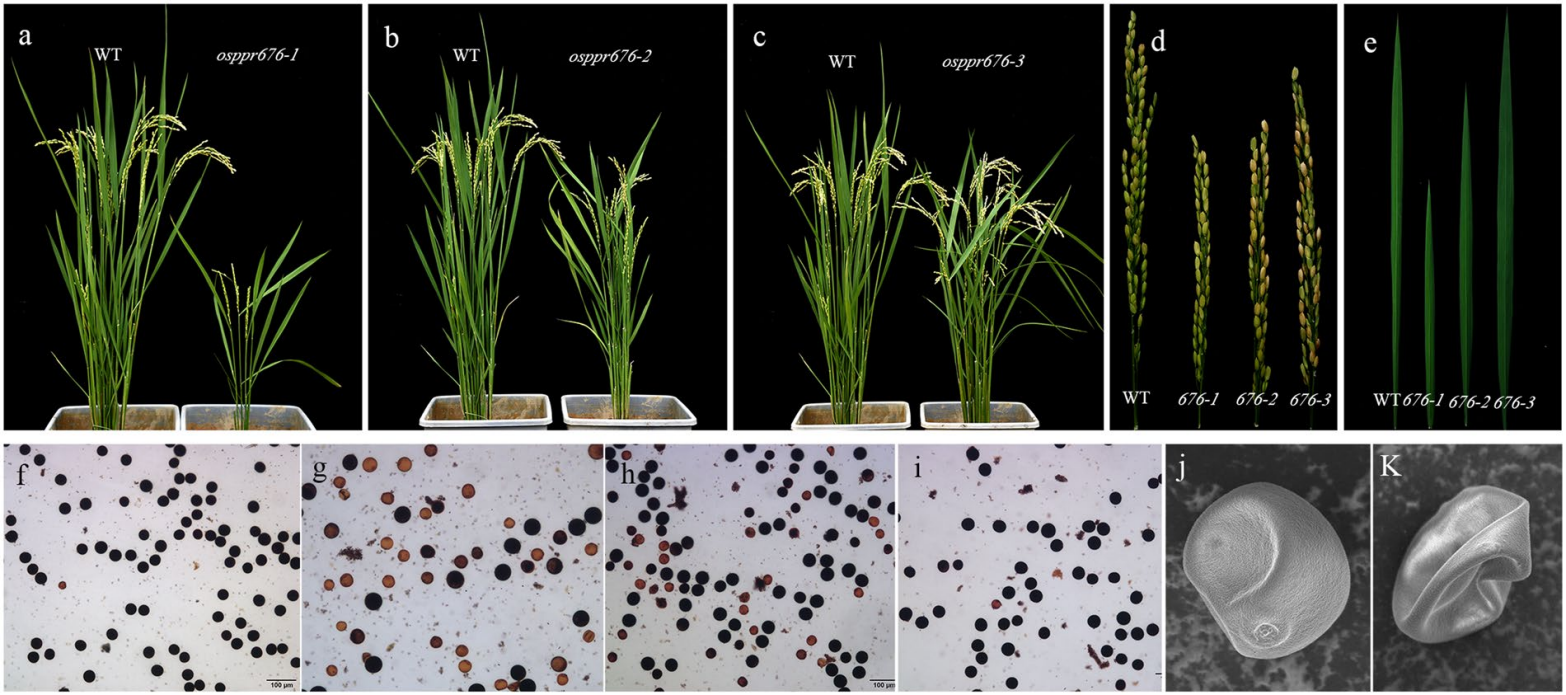
Phenotypes of the *osppr676* alleles. (**a**,**b**) and (**c**) Phenotype comparison of the WT, *osppr676-1, osppr676-2* and *osppr676-3* mutant plants. (**d**) and (**e**) Panicle length and leaf length comparison of the WT, *osppr676-1*, *osppr676-2* and *osppr676-3* mutants. (**f**), (**g**), (**h**), and (**i**) Pollen grain from the WT (**f**), *osppr676-1* (**g**), *osppr676-2* (**h**), and *osppr676-3* (**i**) respectively, stained with iodine potassium iodide solution. Bars = 100 um. (**j**) and (**k**) A higher magnification SEM image of a single pollen grain from WT (**j**) and *osppr676-1* (**k**), respectively.

All *osppr676* mutants were created by the CRISPR/Cas9 system in Nipponbare background (*osppr676-1, osppr676-2, osppr676-3* and similar lines). The truncated protein structures of osppr676-1, osppr676-2, osppr676-3 were shown in Fig. S2. All these mutant alleles displayed different levels of growth retardation and partial pollen sterile phenotypes. In the *osppr676-1* allele, one base insertion in the 3rd PPR motif results in a frame shift and creates a stop codon in the 395^th^ amino acid (8th PPR motif). *Osppr676-1* and all similar lines mutated in the same target sequence showed significant growth retardation, slightly pale green but viable seedlings (Fig. 4a), and partial sterile pollen phenotypes (Fig. 4g). In the *osppr676-2* allele, a two-base deletion induces a frame shift, resulting in changes of amino acids in the 9th PPR motif, and creates a stop codon at the 483^th^ amino acid (10th PPR motif). This allele and similar lines showed a milder phenotype, but still significant growth retardation (Fig. 4b) and partial pollen sterility (Fig. 4h) compared with the WT. This partial pollen sterile phenotype of *osppr676-1* and *osppr676-2* is similar to previously reported *pss1* and *oshub1* ^29, 30^. In the *osppr676-3* allele, one base deletion results in a frame shift, and the translation is terminated by a stop codon in the 580^th^ amino acid. However, *osppr676-3* and WT plants were phenotypically indistinguishable with regard to plant growth and pollen fertility (Fig. 4c and i). Compared with the WT, *osppr676-1* and *osppr676-2* had significantly shorter leaf and panicle, but no any change for *osppr676-3* (Fig. 4d and e). The different phenotypes are attributed to mutations in different motifs, the functions of which will be discussed later.

To further characterize the pollen differences between the WT and the *osppr676-1* mutant, we examined the pollen grains using scanning electron microscopy. Mature WT pollen grains were plump, but only approximately half of the mutant pollen grains had a plump shape similar to the WT pollen, while the other half had a severe shrunken phenotype (Fig. 4k).

**Figure 5:**
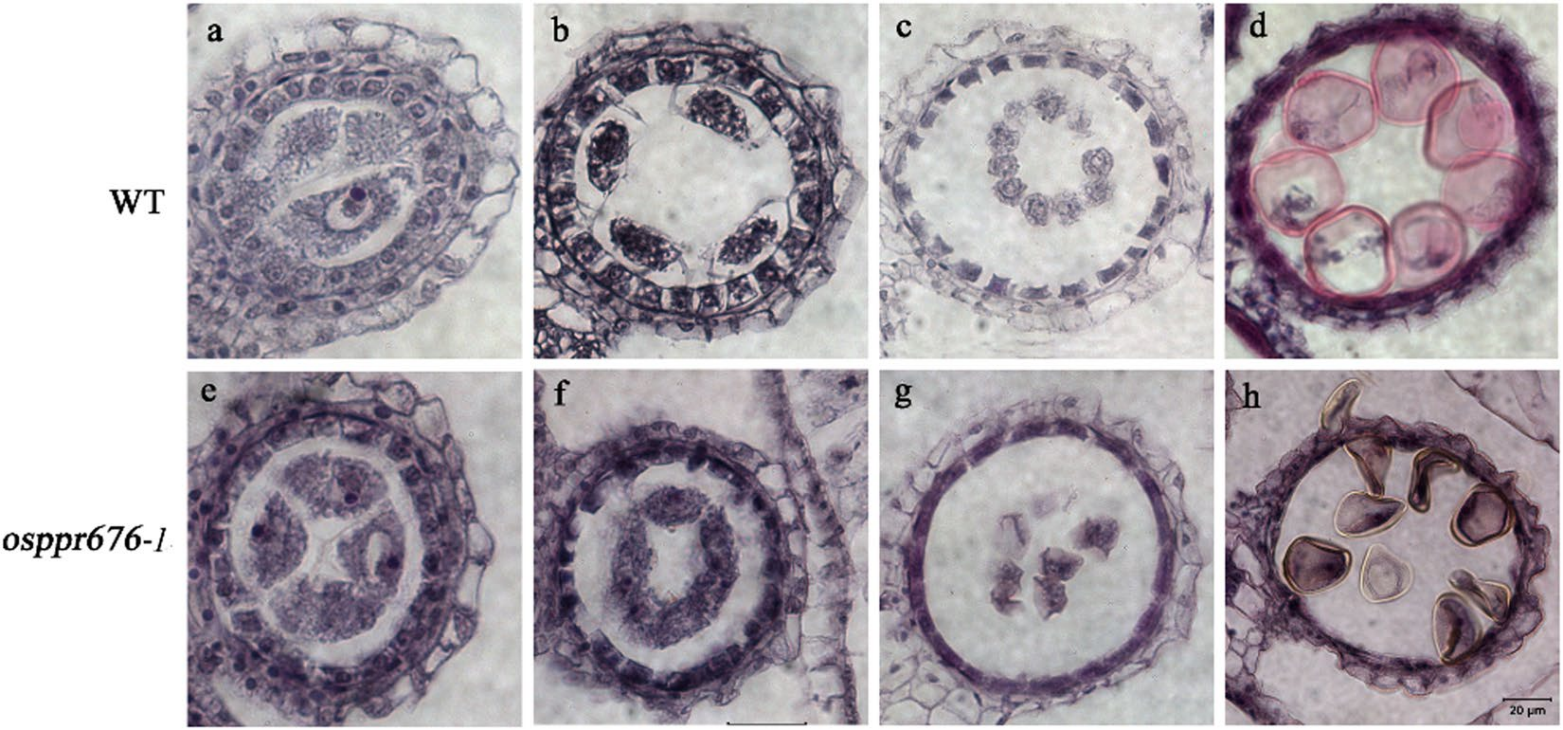
Comparison of cross sections of anthers from WT and *osppr676-1* mutants. The WT anther is shown in (**a**,**b**,**c**), and (**d**) and the *osppr676-1* mutant anther in (**e**,**f**,**g**), and (**h**). (**a**) and (**e**) The MMC (microspore mother cell) stage. (**b**) and (**f**) The late meiosis stage. (**c**) and (**g**) The young microspore stage. (**d**) and (**h**) The vacuolated pollen stage. Bars = 20 um.

To more precisely determine the timing of the anther developmental defects in *osppr676* mutants, we observed their anther sections at different developmental stages under light microscopy (Fig. 5 and Fig. S3). The WT anther sections were used as control. The results revealed that there were no significant differences between WT and *osppr676-1* before the vacuolated pollen stage (Fig. 5). Once the anther developed to the vacuolated pollen stage, tapetal cells degenerated and produced abundant electron-dense Ubisch bodies. The microspores displayed vacuoles and had a round shape in WT anthers (Fig. 5d). However, in *osppr676-1* anthers, only approximately half of the microspores had a normal round shape, similar to the WT, while the rest of the microspores were shrunken and irregularly shaped (Fig. 5h). These results are consistent with the pollen staining (Fig. 4g) and SEM data (Fig. 4k), indicating that *osppr676-1* has partial pollen sterility. For *osppr676-2* and *osppr676-3*, no significant differences were observed comparing with WT during all examined anther developmental stages (Fig. S3).

**Figure 6:**
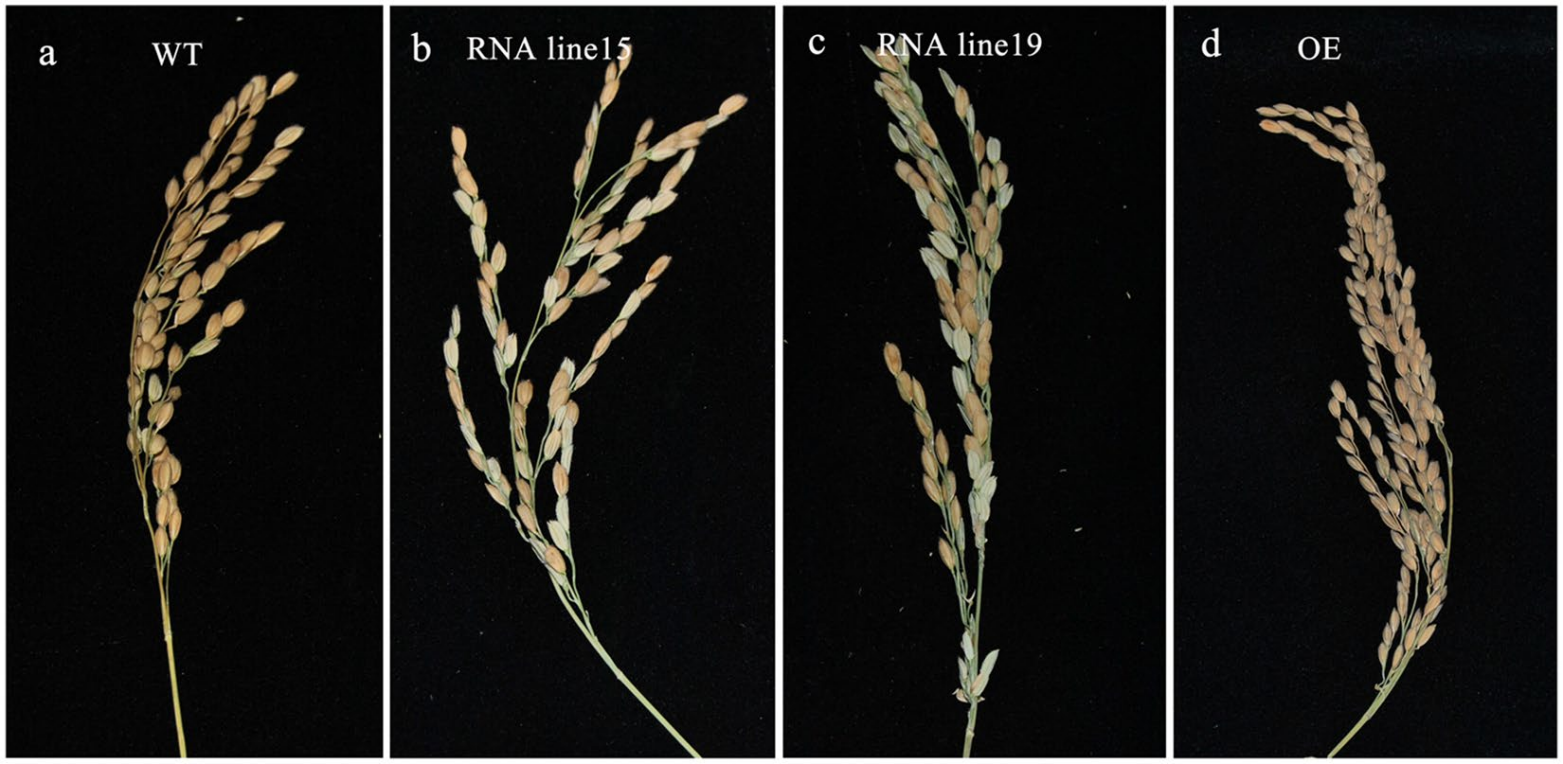
Phenotypes of the transgenic *OsPPR676* RNAi and over expression lines. (**a**) to (**d**) Comparison of WT panicle (**a**), *OsPPR676* RNAi line15 (**b**) and line19 panicle (**c**), and OsPPR676 over expression line (**d**) at the harvest stage.

*OsPPR676* RNAi and over-expression (OE) transgenic rice lines were also generated. As expected, the pollen fertility was declined in both RNAi line15 and 19, which resulted in reduction of their seed-setting rates from 84% of WT to ~47% and ~68%, respectively (Fig. 6). However, the low seed-setting rate did not appear on the transgenic OE lines (Fig. 6; Table S4). Therefore, all above results consistently indicate that OsPPR676 determines the fertility of pollen in a large extent.

### Translation of *atpB* mRNA is disrupted in the mutants

**Figure 7:**
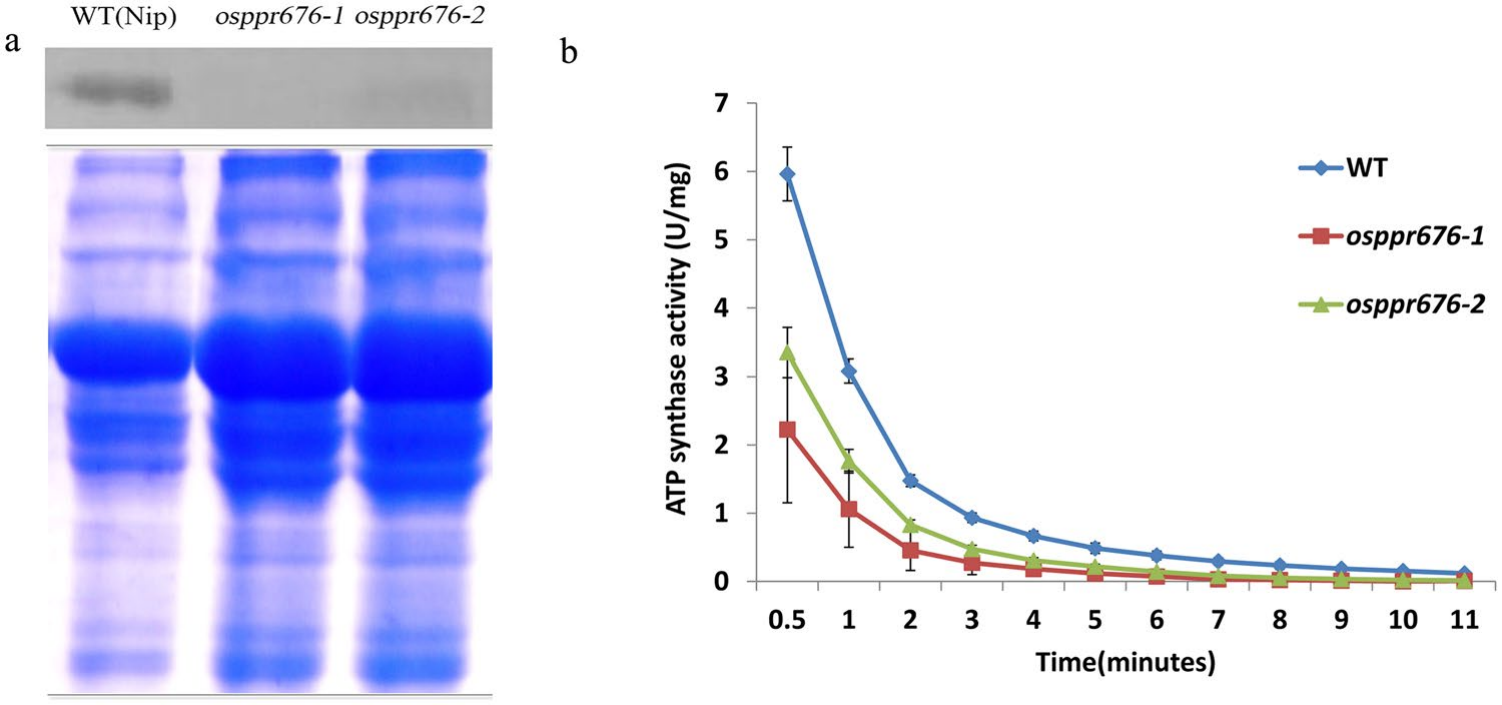
Absence of AtpB subunit production and reduction of ATP synthase activity in rice *osppr676* mutants. (**a**) Western blot analysis with AtpB antibody on total proteins from the *osppr676-1* and *osppr676-2* mutants and WT seedlings (Up panel) and Coomassie Blue staining of the loading amount of samples (Down panel). Full-length gel and blot with original contrast are displayed in Fig. S6. (**b**) ATP synthase activity measured in the WT, *osppr676-1* and *osppr676-2*.

The question here is how does OsPPR676 determine the fertility of pollen in a large extent? It is known that *atpB* in the chloroplast encodes an important β-subunit of ATP synthase for production of ATP, which is essential for the synthesis of most cell organic components, including fatty acids. In maize, the PPR-SMR protein ATP4 is essential for the translation of this chloroplast *atpB* ^18^. To test whether the rice orthologue OsPPR676 has the similar function with the maize ATP4, we performed western blot analysis with an AtpB antibody to detect AtpB in WT and *osppr676* mutants. The results revealed that the subunit AtpB production of the chloroplast ATP synthase was significant reduction in mutant *osppr676-1 and osppr676-2* (Fig. 7a). A small quantity of AtpB production can be detected at low levels in the osppr676-2 line, in contrast with undetectable AtpB in the osppr676-1 line. This is in congruence with the results of ATP synthase activity(Fig. 7b) and the intermediate phenotype observed (Fig. 4). We also have already checked the RNA editing status of *atpB* and mRNA accumulation of the *atpB*, and we found that they all had no detectable alternation in *osppr676* mutants (Fig. S4). These results thereby indicate that the rice pentatricopeptide repeat-SMR protein OsPPR676 similar to the maize orthologue ATP4 protein is also essential for the translation of rice chloroplast *atpB* subunit.

### ATP synthase activity is significantly declined in the leaf tissue of the *osppr676* mutants

Considering the fact that the AtpB is a subunit of the ATP synthase, reducing or losing the production of this subunit will most likely affect the activity of the holoenzyme. To test this hypothesis, we assayed ATP synthase activity in WT and *osppr676* mutants. The results indicated that the ATP synthase activity in all detected lines was gradually declined during the assay (Fig. 7b). *osppr676-1* and *osppr676-2* mutants all show lower activity than WT at different degrees and time points. For instance, ATP synthase activity of *osppr676-1*, who has the most serious phenotype with the single base insertion mutation in the 3^rd^ PPR, is almost three folds lower than that of WT at 30 sec (Fig. 7b). Furthermore, during the whole process of reaction time extended from 0.5 to 11 min, this reduction is still kept (Fig. 7b). These results hence confirm that the OsPPR676 is really essential for the synthesis of rice AtpB subunit and thus the activity of ATP synthase.

### Generation of fatty acids and starch is altered in anther and pollen of *osppr676* mutants

**Figure 8:**
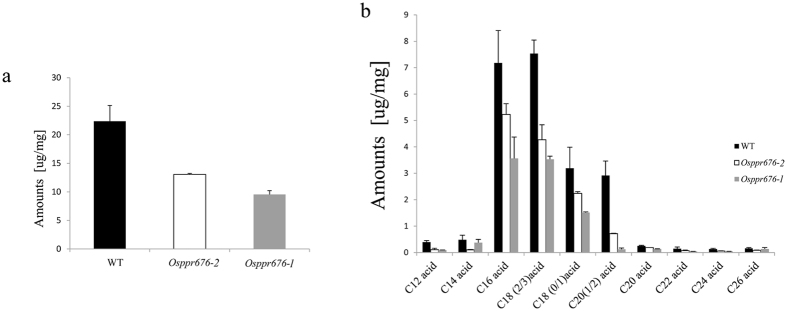
Analysis of anther soluble lipids in the WT, *osppr676-1*, and *osppr676-2*. (**a**) Total soluble lipids amount per mg dry weight anther in the WT (black bars) and *osppr676-1*(gray bars) and *osppr676-2* (white bars). Error bars indicate SD (n = 5). (**b**) Soluble lipids constituents, amount per mg dry weight anther in the WT (black bars) and *osppr676-1*(gray bars) and *osppr676-2* (white bars). Error bars indicate SD (n = 5). Compound names are abbreviated as follows: C12 acid, lauric acid; C14 acid, tetradecanoic acid; C16 acid, hexadecanoic acid; C18 (0/1)) acid, octadecanoic acid and oleic acid; C18 (2/3) acid, linoleic acid and linolenic acid; C20 acids, Eicosanoic acid; C20 (1/2) acid, paullinic acid and eicosadienoic acid; C22 acid, docosanoic acid; C24 acid: tetracosanoic acid; C26 acid, hexacosanoic acid. All acids were analyzed as methyl esters.

Fatty acids and starch are two important components required for normal plant growth and pollen development^31^, which synthesized in the plastids in an ATP-dependent process^31–33^. To determine whether generation of the fatty acid components was altered in the *osppr676* mutants, we analysed the composition of total soluble lipids from WT and *osppr676* anthers using gas chromatography-mass spectrometry (GC-MS) and gas chromatography-flame ionization detection (GC-FID)^34^. The results showed that the total soluble lipids in *osppr676-1* and *osppr676-2* anthers were greatly reduced compared to WT. We detected a concentration of 22.377 μg/mg soluble fatty acids in WT anthers but only 9.541 μg/mg and 13.057 μg/mg in *osppr676-1* and *osppr676-2* (Table S5), respectively, which corresponds to a 57.36% and 41.65% reduction, respectively, compared to WT (Fig. 8a). Although the major fatty acid components were still detected in mutant anthers, almost all components from C12 acid to C26 acid were significantly reduced (Fig. 8b). A detailed description of the altered fatty acid components is shown in Table S5. The altered synthesis of starch component can be seen from Fig. 4g,h, which have 20% to 45% of the iodine-stained pollen grains filling with no or very little starch. All above results suggest that OsPPR676 involves in the biosynthesis of both fatty acids and starch, which are important components required for normal plant growth and pollen development.

## Discussion

### The plastid-localized OsPPR676 is essential for plant growth and pollen development in rice

*OsPPR676* is the ortholog of *ATP4* and *SVR7* and has a similar function for production of AtpB subunit (Fig. 7), but the phenotypic expression of these three genes after mutation is partially different. The *atp4*, which was screened from the collection of nonphotosynthetic maize mutant, exhibits pale-green phenotypes and the homozygous dies after three weeks of growth on soil^18^. The mutations of *SVR7* account only for slightly pale green and developmentally retarded but viable and fertile plants^17, 35^. By contrast, the CRISPR/cas9-induced mutants *osppr676-1 and osppr676-2* created in this study display viable, developmentally retarded and partially sterile plants (Figs 4 and 5h). These phenotypic variations were attributed to the differences in the accumulation of ATP synthase subunits as suggested by Zoschkel *et al*. (2013) previously. As long as accumulation of one or more ATP synthase subunits is reduced, the activity of ATP synthase and ATP yield will be depressed. This will affect ATP-dependent biosynthesis of fatty acids, carbohydrates, and other organic matters, finally impacting on plant growth and pollen development. Following such a consideration, we measured ATP synthase activity of WT and *osppr676* mutants. The data revealed it was indeed remarkably reduced in two severe mutants *osppr676-1* and *osppr676-2* (Fig. 7b). We further analyzed composition of total soluble lipids in the anthers of these *osppr676* mutants. The results, as expected, confirmed the great reduction of total soluble lipids in *osppr676-1* and *osppr676-2* as compared with that in WT (Fig. 8). In addition, the retarded growth of the plants (Fig. 4a,b) and appearance of the sterile pollens with faint iodine-staining (Fig. 4g,h) also indicates the significant reduction of the total dry matter including carbohydrates, proteins, and lipids that were synthesized in these mutant plants.

Lipids play crucial roles in successful pollen development^31^. Fatty acids and carotenoids are the major components of pollen coat, which are deposited from outside of the pollen, as they are synthesized and secreted by the tapetum^36^. By far, several genes involved in synthesis and transport of these tapetal lipids have been confirmed essential for pollen fertility, such as *Wax-deficient anther 1*(*WDA1*)^37^, *Defective pollen wall* (*DPW*)^38^, *CYP704B2* ^39^, and *Fax1* ^40^. Furthermore, polar lipids involve in the form of densely packed membranes, and neutral lipids such as triacylglycerols (TAG) involve in the form of lipid droplets^41, 42^. Starch also accumulates as internal substances of pollen during development. Mutation of the genes that are responsible for the synthesis and transport of these internal lipids and carbohydrates also results in defects of male reproduction, such as mutant *gpat9* ^43^, *iput* ^44^, *hmg2* ^45^, *smt2* ^46^, *csa* ^47^, and *mia* ^48^. Datta *et al*.^32^ found that two proteins, hexose transporter and plasma membrane H-ATPase, involving in starch biosynthesis, altered patterns of expression during pollen maturation process and determined pollen sterility. All these reports provide the supporting evidences that *OsPPR676* plays crucial role in the ATP-dependent biosynthesis of organic matter such as lipids, proteins, and carbohydrates via regulating production of AtpB subunit of ATP synthase, thus affecting plant growth and pollen development in rice.

### The SMR domain is not essential and PPR motifs are important for OsPPR676 protein function

To study the functions of the OsPPR676 motifs, we designed three different target sequences of the CRISPR/Cas9 mutation system, resulting in different mutant lines with a disruption in different regions of the 3′-domain of OsPPR676. In the *osppr676-3* allele, the mutation produces a truncated OsPPR676 protein with a disrupted SMR domain (Fig. S2). Several PPR-SMR proteins have been analysed experimentally thus far^15, 17–20, 49^, but the function of the SMR domain in PPR proteins is still unknown. In this study, *osppr676-3* and WT plants were phenotypically indistinguishable with regard to plant growth and pollen fertility (Fig. 4c,i), which indicates that the SMR domain is not essential for OsPPR676 function. The SMR domain was originally identified as the C-terminal region of bacterial MutS2 proteins^19^. Although the SMR domain is believed to function in transcription and to have DNA-binding activity, there is little experimental data on its contribution to PPR-SMR function. In higher plants, PPR-SMRs are a very small subgroup of PPR proteins, with only 8 members in *Arabidopsis* ^13^. Most reported molecular functions of the SMR domain are from bacteria and mammals^50–53^. The only exception is GUN1 from *Arabidopsis*, but all *gun1* mutants showed wild type-like phenotypes under normal growth conditions^54^. Although the present findings provide a foundation for hypotheses concerning the functions of the SMR domain in PPR-SMR proteins, an alternative possibility is that, as an ancient domain, the function of the SMR domain has been degraded in plant PPR-SMRs during evolution.

The last two PPR motifs and the SMR domain are destroyed in the *osppr676-2* allele mutant (Fig. S2). Phenotypic analysis indicated that the *osppr676-2* mutants showed a milder phenotype than *osppr676-1*, but still significant growth retardation (Fig. 4b) and partial pollen sterility (Fig. 4h) compared with the WT. These results indicated that the last two PPR motifs are important for OsPPR676 function. The truncated version of OsPPR676 may also be produced in the *osppr676-1* mutant, which has an amino acid change from 3rd PPR motif, and the last three PPR motifs and the SMR domain are completely lost (Fig. S2). The *osppr676-1* mutant has a much more serious defective phenotype, showing very weak growth with shorter and fewer spikelets than WT plants, and its pollen sterility is also more severe than *osppr676-2* (Fig. 4a and g). These results indicate that the major functions of OsPPR676 are destroyed in *osppr676-1*, and the third to eighth PPR motifs play essential roles in OsPPR676 function.

Therefore, the SMR domain of OsPPR676 is not critical for the function in the Nipponbare background, but PPR motifs can not be destroyed for normal protein functions. In addition, different target sequences can be selected according to the purposes of the study using the CRISPR/Cas9 mutation system to generate multiple mutants (Table S2). Reliable phenotypic analysis and functional studies of motifs could be supported by many diverse mutants. Thus, the CRISPR/Cas9 mutation system is a useful tool to study the role of different protein motifs.

### OsPPR676 and Osj10gBTF3 are likely involved in the same male reproduction regulation pathway

In our previous study, the candidate protein OsPPR676 was shown to interact with Osj10gBTF3^21^. The interaction of these two proteins was confirmed by bimolecular fluorescence complementation (BiFC) assays in this study (Fig. 2). *BTF3* encodes the β-subunit of the nascent polypeptide-associated complex (NAC), which not only plays a role in the nucleus, influencing the transcription levels of some genes^55–57^, but is also involved in the targeting and translocation of nascent polypeptides^58–60^. Repression of Osj10gBTF3 causes significant plant miniaturization and pollen abortion^21^, which are similar to the phenotype of *osppr676*-*1*, although phenotype severity is different. Therefore, we hypothesize that Osj10gBTF3 probably participates in OsPPR676 transportation to the plastids. Although Osj10gBTF3 and OsPPR676 are not localized in the same organelle, they most probably interact in the cytoplasm during the transportation of OsPPR676 from cytoplasm, where it is produced, to plastids. Consequently, OsPPR676 and Osj10gBTF3 may be involved in the same male reproduction regulatory pathway. Further studies are still needed to confirm this hypothesis.

## Materials and Methods

### Plant materials and growth conditions

The *osppr676* mutants were generated by the CRISPR/Cas9 mutation system on the Nipponbare background (*Oryza sativa L. ssp. japonica*). The sgRNA-CRISPR/Cas9 plant expression vectors were constructed (Fig. S1), and rice transformation was performed as previously described^61^. PCR was carried out to amplify the genomic regions surrounding the CRISPR target sites using specific primers (Table S1) to confirm the mutants. The confirmed T_0_ mutants were initially cultivated in the growth chamber and then transplanted into paddy field to grow to mature.

### Pollen fertility examination

To analyse pollen viability, pollen sampled from the spikelets of the WT and mutant plants just before flowering was stained with 1% (w/v) iodine and potassium iodide (I_2_–KI) solution^62^. Three biological replicates were used. The stained pollen grains were visualized, and images were recorded using a Leica DMIRB fluorescence microscope.

### Generation of OsPPR676 over-expression and RNAi lines

To construct the over-expression vector, the *OsPPR676* cDNA fragment was PCR amplified with primers OsPPR676-OE-F and OsPPR676-OE-R (Table S1), and ligated into the binary vector pCAMBIA1300 to produce the over-expression construct *pCAMBIA1300-35S::OsPPR676::NOS*. For the RNAi vector construction, the PCR products amplified from cDNA using the primers OsPPR676-KpnI and OsPPR676-BamHI, OsPPR676-SpeI and OsPPR676-SacI (Table S1) were digested with two pairs of restriction enzymes: KpnI/BamHI and SpeI/SacI, respectively. The different digested fragments were then ligated into the binary vector pTCK303. Both vectors were introduced into the wild-type cultivar Nipponbare using *Agrobacterium*-mediated transformation. Transformation was performed as previously described^61^.

### Scanning electron microscopy analysis

Fresh WT (Nipponbare) and *osppr676-1* mutant pollen grains were air dried for approximately 30 minutes before viewing on a Hitachi S-4700 scanning electron microscope at an accelerating voltage of 5 kV.

### Light microscopy of cytological sections

The developing stages of anthers were classified according to spikelet length and anther morphology^63^, and anthers, leave and stems were then collected and fixed in a solution containing 50% ethanol, 5% glacial acetic acid, and 3.7% formaldehyde for 24 h at 4 °C and were dehydrated using an ethanol series (50%, 70%, 85%, 95%, and 100% ethanol). After clearing with xylene and paraffin wax infiltration, the sample was embedded and sectioned at 6 to 10 µm thickness under a Leica 2035 Biocut. The sections were stained with haematoxylin and 0.5% eosin for anthers or 0.5% toluidine blue for leave and stems. Images were captured using a Nikon Eclipse 80i microscope.

### Analysis of anther total soluble lipids

Soluble lipids of the anthers were analysed as described previously^37–39^. To extract soluble fatty acids, 10 mg of freeze-dried anther materials were extracted with 1 mL of chloroform/methanol (1:1 [v/v]). They were first incubated at 50 °C for 30 min and then overnight with constant shaking at room temperature. This extraction was repeated three times to ensure that no soluble lipids were left in the anther samples. The combined lipid extracts were then evaporated under a gentle stream of nitrogen gas until they reached a final volume of 100 μL. All soluble lipid samples were transesterified in 1 mL of 1 N methanolic HCl for 2 h at 80 °C. After the addition of 2 mL of saturated NaCl/water and 20 μg of dotriacontane (Fluka) as an internal standard, the transesterified soluble lipids were subsequently extracted three times with 1 mL of hexane. The organic phases were combined, the solvent evaporated, and the remaining sample derivatized as described above. GC-MS and GC-FID analyses were performed as described by Franke *et al*.^34^. The results of soluble lipid analysis were related to dry weights of the anthers.

### Subcellular localization of the OsPPR676 protein

To generate a translational protein fusion between the OsPPR676 signal peptide and GFP, full-length *OsPPR676* was amplified by PCR from WT rice (Nipponbare) and cloned into pENTR/D-TOPO (Invitrogen, USA). The fusion was introduced into the binary vector pGWB5 (a gift from Tsuyoshi Nakagawa, Shimane University) by GATEWAY site-specific recombination (Invitrogen). The binary construct contains a cauliflower mosaic virus 35 S promoter that allows constitutive expression of the full-length OsPPR676: GFP fusion. Then, these constructs were transformed into *Agrobacterium tumefaciens* strain EHA105. Using established protocols^64^, the EHA105 strain harbouring the OsPPR676: GFP fusion expression plasmid was infiltrated into *Nicotiana tabacum* leaves to transiently express the fusion protein.

In addition, rice protoplasts were isolated from etiolated stems of 9-day-old Zhong Hua11 grown at 28 °C in a dark chamber. The pGWB5*-OsPPR676: GFP* plasmid and the mitochondrial marker *F1-ATPase- γ:RFP* were co-transformed into rice protoplasts by polyethylene glycol–mediated transformation as previously described^65^. The infiltrated *Nicotiana tabacum* leaves and rice protoplasts were used for GFP and MitoTracker red signal detection by an Olympus FluoView FV1000 confocal microscope.

### RNA *in situ* hybridization

To generate gene-specific and control sense RNA probes, we amplified the gene-specific region of the *OsPPR676* cDNA fragment using primers listed in Table S1, which was then cloned into the pGEM-T vector (Promega). The clone was digested with SalI and transcribed *in vitro* under the T7 and SP6 promoters by RNA polymerase using a DIG RNA labelling kit (Roche). *In situ* hybridization was performed as previously described^66, 67^.

### qRT-PCR analysis

Total RNA was isolated using TRIzol reagent from rice tissues, including the root, stem, leaf, sheath, anther, pistil, palea, lemma, and anther at different stages according to spikelet length and anther morphology^63^. Approximately 1 μg of RNA per sample was treated by genomic DNA eraser (TaKaRa) and then used to synthesize cDNA with the PrimeScript RT reagent kit. Two microlitres of cDNA was used as a template for RT-PCR. All the primers for RT-PCR are listed in Table S1. Real-time quantitative PCR was performed with a LightCycler 480 (Roche). Amplification was conducted using the following protocol: 95 °C for 30 s, 40 cycles of 95 °C for 5 s, and 60 °C for 30 s. *ACTIN* was used as an internal control. Each experiment was repeated three times, each with three replicates.

### Bimolecular fluorescence complementation assays

By applying Gateway technology, the full-length cDNAs of *OsPPR676* and *Osj10gBTF3* were cloned in binary pBiFC vectors containing the amino-terminal fragment of the eYFP fluorescent protein or carboxy-terminal fragment of the eCFP fluorescent protein (eYFP^N^ and eCFP^C^, respectively)^24^. CFP^C^ is modified from YFP^C^ to enhance the fluorescence signal in BiFC assays. All constructs were transformed into EHA105. Different combinations of these constructs were mixed at a 1:1 OD_600_ ratio and injected into 3–4-week-old tobacco epidermal cells. After 36 hours, fluorescence signals were observed under a confocal fluorescence microscope.

### Western-Blot Analysis

Total leaf proteins of wild-type, *osppr676-1* and *osppr676-2* plants were extracted as described previously^68^, and then subjected to SDS-PAGE on 12% gels. The quantification was confirmed by Coomassie Blue staining of the six different samples. For western-blot analysis, proteins on gels were electrophoretically transferred to a polyvinylidene difluoride membrane and blocked with a blocking buffer (3% bovine serum albumin in phosphatebuffered saline [PBS] buffer [20 mM Tris-Cl, pH 7.5, and 150 mM NaCl]). After washing with PBS buffer, the membrane was incubated with a polyclonalantibody against AtpB (1:2,000 dilution) in the blocking buffer for 10 h 4 °C. After washing with PBS buffer and then incubating for 1 h with goat anti-rabbit IgG-conjugated alkaline phosphatase (1:2,000 dilution). The antibody was obtained from Agrisera (AtpB).

### Measurement of ATP synthase activity

The protein were prepared from 200 mg fresh leaf sample and measured by continuous NADH-coupled enzyme assay kit (Genmed 50250.5, Shanghai, China). The measurement was typically performed at 28 °C with 0.05 ml sample protein in 0.95 ml volume of the ATP synthase reaction buffer supplemented with a regeneration system (3 mM PEP, 20 U/ml PK), 20 U/ml LDH and NADH. Absorbance data are collected at A340 using a SPECTRAmax 250 microplate spectrophotometer equipped with SOFTmax PRO software (Molecular Devices). Each line of WT and *osppr676* mutants was measured with three biological replicates.

### Data availability

Sequence data from this article for the cDNA and genomic DNA of OsPPR676 and Osj10gBTF3 can be found in the GenBank/EMBL/Gramene data libraries under accession numbers LOC_Os03g1167 0 and LOC_Os10g34180 respectively.

## Electronic supplementary material

Supplemental data
