# Supplementary material for: A Plastid-Localized Pentatricopeptide Repeat Protein is Required for Both Pollen Development and Plant Growth in Rice
Source: Sci Rep. 2017 Sep 13;7:11484. doi: 10.1038/s41598-017-10727-x (PMC5597598; doi:10.1038/s41598-017-10727-x)
Supplement: Supplementary file 1 — Supplemental data [file 41598_2017_10727_MOESM1_ESM.pdf]

A Plastid-Localized Pentatricopeptide Repeat Protein Is Required for Both Pollen Development and Plant Growth in Rice

Yu-Jun Liu[a,b,‡], Xuejiao Liu[a,‡], Chen Hao[a], Peng Zheng[a], Wenyi Wang[a], Liangchao Wang[a], Jianhua Zhang[b,*], Jumin Tu [a,*]

[a]Institute of Crop Science, College of Agriculture and Biotechnology, Zhejiang University, Hangzhou 310058, PR China
[b]State Key Lab of Agrobiotechnology, School of Life Science, The Chinese University of Hong Kong, N.T. Hong Kong
[‡]These authors contributed equally to this work.

[*]Corresponding Authors:
Jianhua Zhang, Rm G88, Science Centre South Block, CUHK, N.T. Hong Kong. E-mail: jhzhang@cuhk.edu.cn Tel: 852-3943-6288
Jumin Tu, Yu-Hang-Tang Rd. 866, Xihu District, Hangzhou 310058, PR China. E-mail: jtu@zju.edu.cn Tel: 86-8898-2835

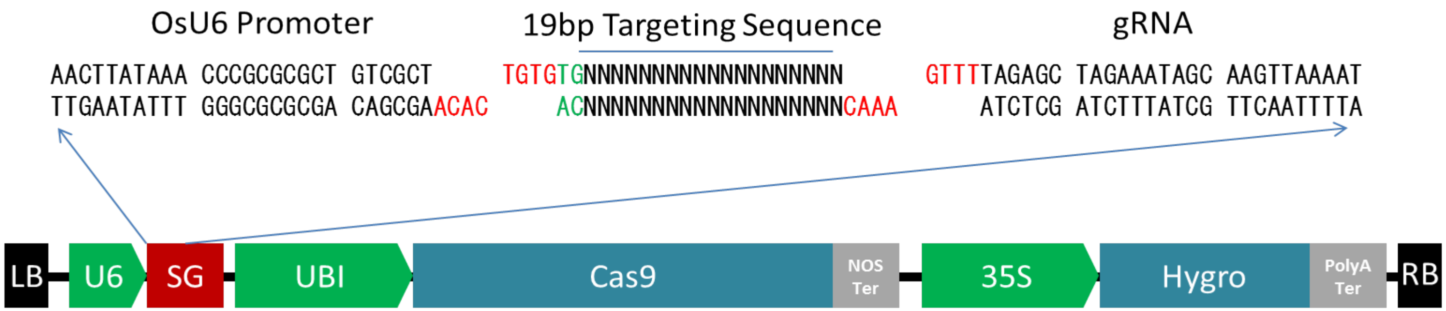

Feature of plasmid

**LB**	Left border of T-DNA	**Cas9**	Optimized Cas9
**RB**	Right border of T-DNA	**NOS Ter**	NOS terminator
**U6**	Rice U6 promoter	**35S**	CaMV 35S promoter
**SG**	sgRNA	**Hygro**	Hygromycin selection marker
**UBI**	UBI promoter	**PolyA Ter**	PolyA terminator

Figure S1. sgRNA-CRISPR/Cas9 plant expression vector.

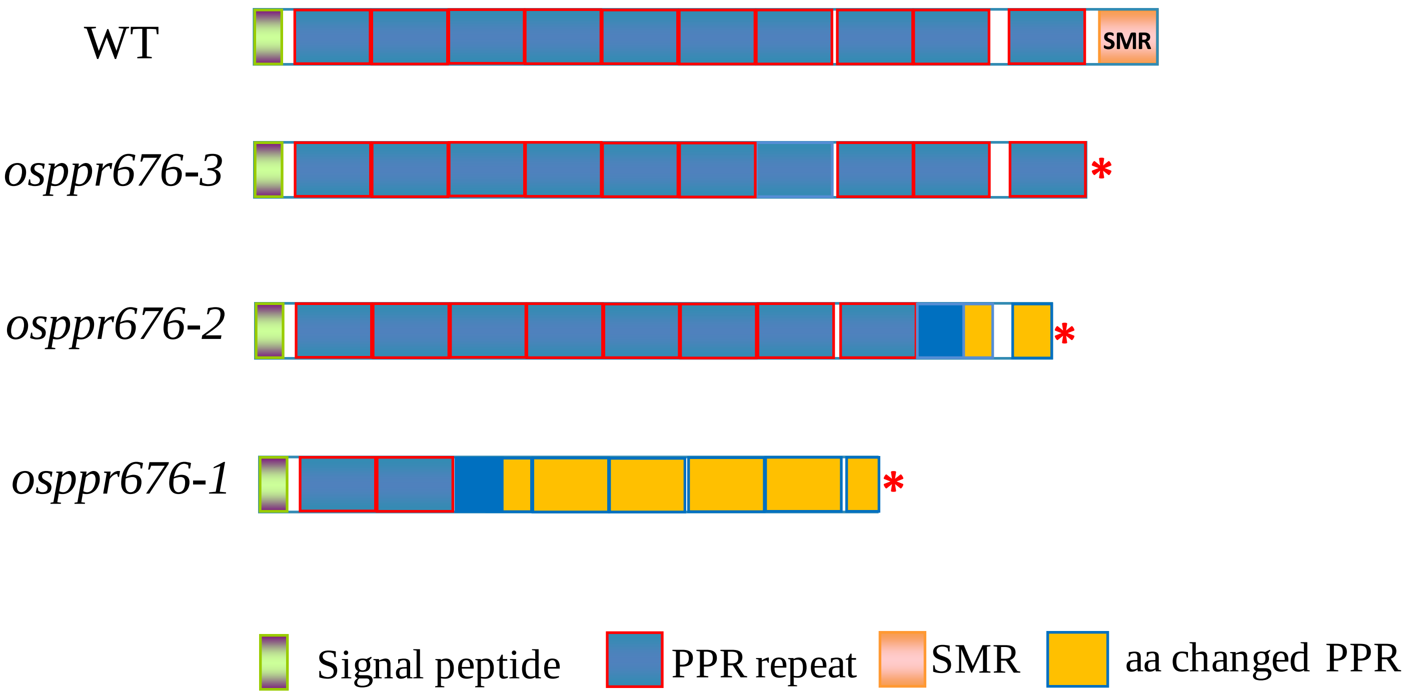

Figure S2. Truncated proteins structure of *osppr676-1*, *osppr676-2*, *osppr676-3*.

.

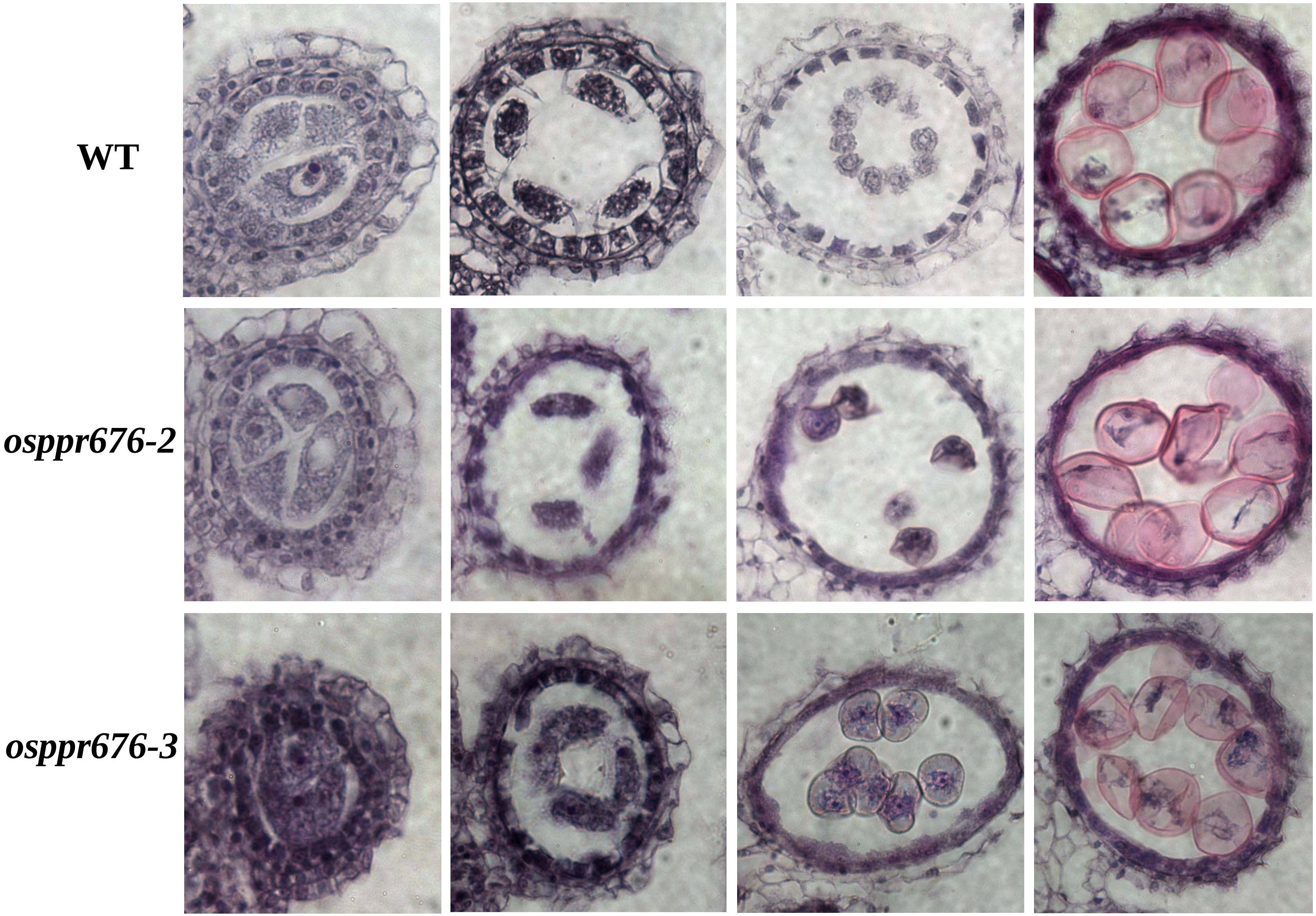

Figure S3. Comparison of cross sections of anthers from wild type and *osppr676-2* and *osppr676-3* mutants.

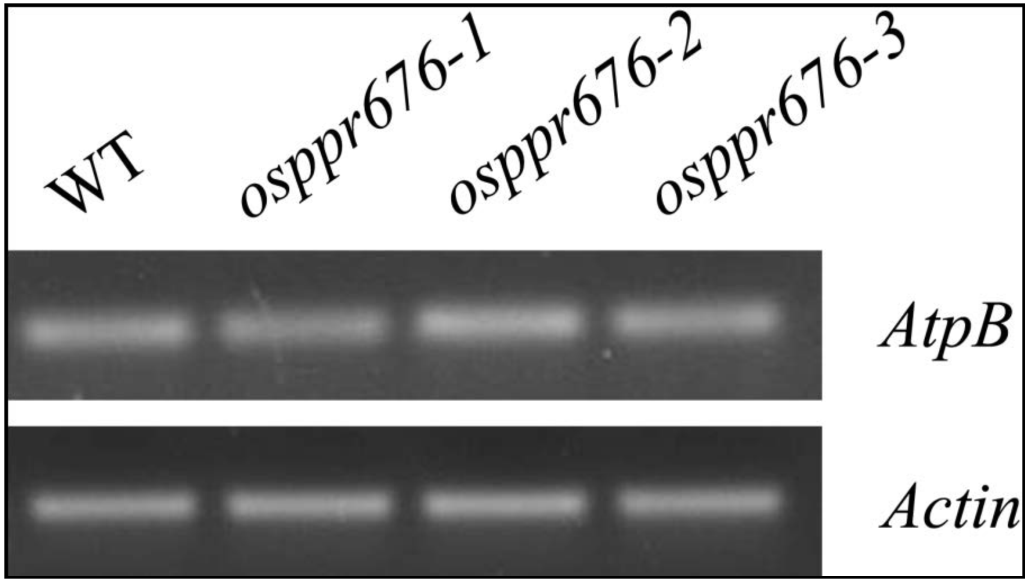

Figure S4. No significant difference was detected in mRNA accumulation of *atpB* in WT and *osppr676* mutants.

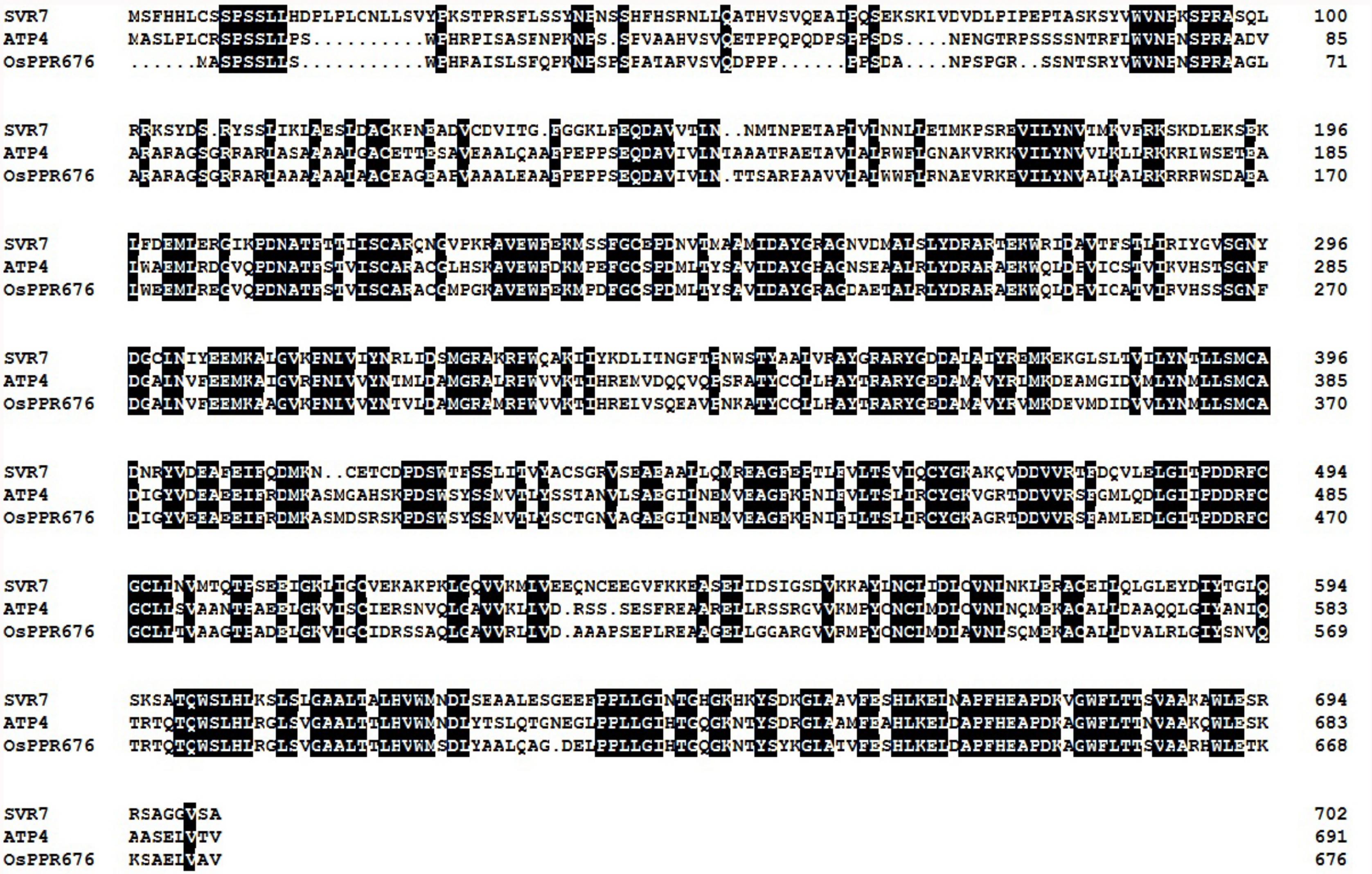

Figure S5. Protein sequences alignment of SVR7, ATP4 and OsPPR676.

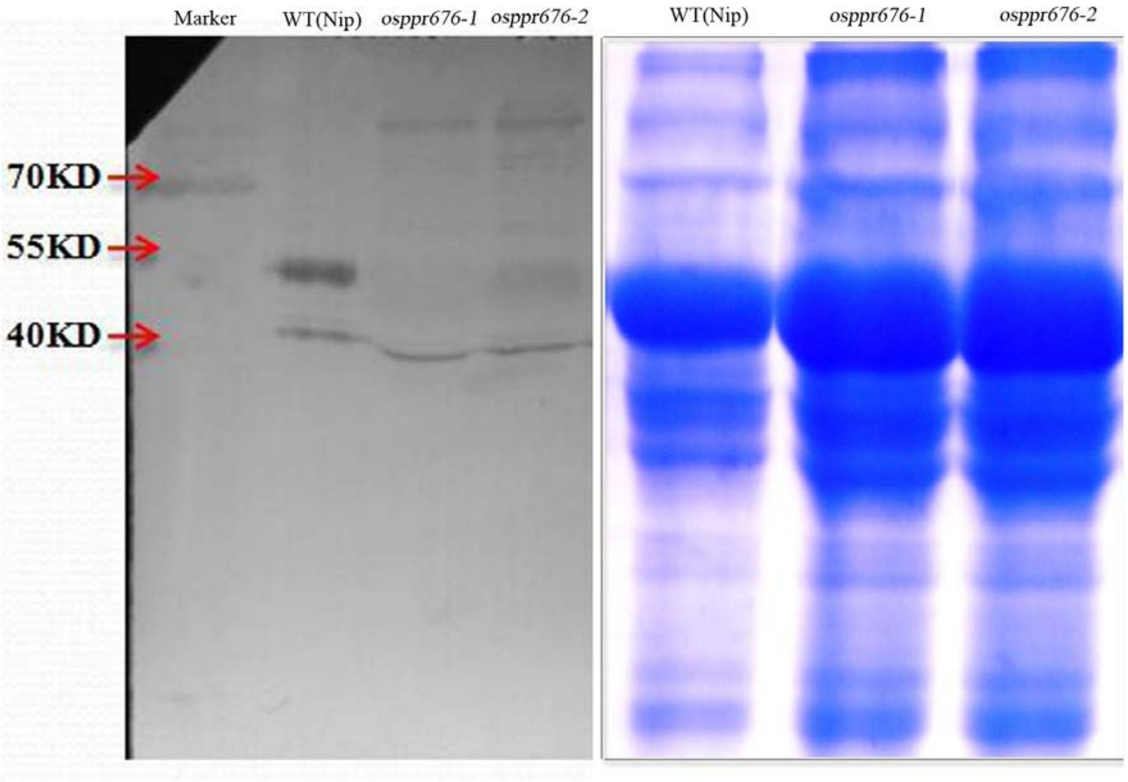

Figure S6. Absence of AtpB subunit production in rice *osppr676* mutants. Western blot analysis with AtpB antibody on total proteins from the *osppr676-1* and *osppr676-2* mutants and WT seedlings and Coomassie Blue staining of the loading amount of samples. The size of target atpB band is 54KD, and these bands about 40KD are unspecific bands.

Table S1. Primers used in this study.

Primer name	Primer Sequence (5'–3')	Use for
OsPPR676 MF	CTCAACGTGTTCGAGGAAAT	Confirm *osppr676-1* mutant
OsPPR676 MR	CAGTTGCAGTACGGCATCCT	Confirm *osppr676-1* mutant
GT638-F	CACCTTCTCGACCGTCATCA	Confirm *osppr676-2* mutant
GT638-R	CGCCTTCATTTCCTCGAACA	Confirm *osppr676-2* mutant
GT640-F	CCTCACCTCGCTCATCCGTT	Confirm *osppr676-3* and *676-4* mutant
GT640-R	AGCAGCGGCGGCAGCTCGT	Confirm *osppr676-3* and *676-4* mutant
OsPPR676-ENF1	CACCATGGCTTCCCCTTCCTCCCT	Construct pENTR vector
OsPPR676-ENR1	CACCGCTACTAACTCTGCCG	Construct pENTR vector
OsBTF3-ENF1	CACCATGAATGTTGACAAGCTCAA	Construct pENTR vector
OsBTF3-ENR1	TGATTCCTTTTTCTCTTCTG	Construct pENTR vector
OsPPR676 probe F	GAGGACGCCATGGCCGTG	RNA in situ hybridyzation
OsPPR676 probe R	TCACCATGGATGAGTAGC	RNA in situ hybridyzation
OsPPR676 RF1	GACCATCCACAGGGAGCTAGT	Real-time PCR
OsPPR676 RR1	ACAAGGCGACGTACTGCTG	Real-time PCR
OsPPR676- OE-F	CTAGTCTAGACAGAGAGAGCATCGCGC	Construct over-expression vector
OsPPR676- OE-R	CGGGGTACCCCATTGTTGCGTTTTACAC	Construct over-expression vector
OsPPR676- KpnI	GATCGGTACCGGATGACCTCCTTGCGAACCTC	Construct RNAi vector
OsPPR676-BamHI	GATCGGATCCCGCCCGTGTCTCCGTCCAA	Construct RNAi vector
OsPPR676- SpeI	GATCACTAGTGGATGACCTCCTTGCGAACCTC	Construct RNAi vector
OsPPR676- SacI	GATCGAGCTCCGCCCGTGTCTCCGTCCAA	Construct RNAi vector

Table S2. Multiple *osppr676* lines created by CRISPR/Cas9 mutation system.

Mutation site	Mutation type
***OsPPR676* SG638 site**	
GGTGCTCGCCGGACATGCTCACGTACT-CGGCGGTGATCGACGC	WT
GGTGCTCGCCGGACATGCTCACGTACC-CGGCGGTGATCGACGC	D1, +1
GGTGCTCGCCGGACATGCTCACGTACTTCGGCGGTGATCGACGC	+1
GGTGCTCGCCGGACATGCTCACGTCCT-CGGCGGTGATCGACGC	D1, +1
GGTGCTCGCCGGACATGCTCACGT----CGGCGGTGATCGACGC	D3
GGTGCTCGCCGGACATGCTCAGGTA---CGGCGGTGATCGACGC	D6, +4
GGTGCTCGCCGGACATGCTCACGTACT-CGGGGGTGATCGACGC	D1, +1
GGTGCTCGCCGGACATGCTCACGTA---CGGCGGTGATCGACGC	D2
GGTGCTCGCCGGACATGCTCACGTC----GGCGGTGATCGACGC	D4, +1

***OsPPR676* SG640 site**

CGTCCGGGGTGATGCCGAGGTCCT-CGA-GCATGGCGAATGACCT	WT
CGTCCGGGGTGATGCCGAGGTGCTCAAT-GCATGGCGAATGACCT	D6, +7
CGTCCGGGGTGCCGCAGAGGTCCT-CAA-GAATGGCGAATGACCT	D18, +18
CGTCCGGGGTGATGCCGAGGTGCT-CAATGCCTGGCGAATGACCT	D10, +10
CGTCCGGGGTGATGCCGAGGTCCT-C---GCATGGCGAATGACCT	D2
CGTCCGGGGTGATGCCGAGGTCCT-CGAAGCATGGCGAATGACCT	+1
CGTCCGGGGTGATGCCGAGGTCC----A-GCATGGCGAATGACCT	D3
CGTCCGGGGTGATGCCGAGGTC-------GCATGGCGAATGACCT	D5
CGTCCGGGGTGATGCCGAGGTCCT-CGA--TATGGCGAATGACCT	D2, +1

***OsPPR676* SG641 site**

GGACGCAGACGCAGTGGTCGCTGCACCTGAGGGGCCTCTCCGT	WT
GGACGCAGACGCAGTGGTCGCTGCACC-GAGGGGCCTCTCCGT	D1
GGACGCAGACGCAGTGGTCGTTG---GTGAGGGGCCTCTCCGT	D7, +4

GGGGCCTCACCGTCGGGTCCGTGC---TGAGGGGCCTCTCCGT	D25, +22
GGACGCAGACGCAGTGGTCGCTGCAC-TGAGGGGCCTCTCCGT	D1
GGACGCAGACGCAGTGGTCGCTGTAC-TGAGGGGCCTCTCCGT	D3, +2
GGACGCAGACGCAGT-----------------GGCCTCTCCGT	D17
GGACGCAGACGCAGTGGTCGCTGCGC-TGAGGGGCCTCTCCGT	D3, +2
GG------------------------------GGCCTCTCCGT	D30
GGACGCAGACGCAGTGGTCGCTGCAT-GGAGGGGCCTCTCCGT	D3, +2

The wild type sequence is shown at the top with the PAM sequence highlighted in brown and the target sequence in blue. Red dashes, deleted bases; red bases, insertions or mutations. Green sequences, *osppr676-1* mutation; yellow sequences, *osppr676-2* mutation; purple sequences, *osppr676-3* mutation. The net change in length is to the right of each sequence (+, insertion; D, deletion). Note that some alterations have both sequence insertions and deletions.

Table S3. Predicted possible off-target sites of SG638, SG640 and SG641 by CRISPR-P (http://cbi.hzau.edu.cn/crispr/).

SG638 postion: Chr3:-6090845 Guide sequence: GGACATGCTCACGTACTCGGCGG

Predicted off-target sites for SG638 site:

Sequence	Score	MMs	Locus	Gene	Region
GGACTTTGCCACGTACTCGGGGG	0.6	4MMs	Chr12:-2141485	LOC_Os12g04960	exon
GGTCATCCGCAGGTACTCGGCGG	0.3	4MMs	Chr1:+33066673	LOC_Os01g57230	exon
CGACATGCCCACGCACACGGCGG	0.1	4MMs	Chr11:-3818498	LOC_Os11g07530	exon
GGTCAGGCTCAAGTGCTCGGTGG	0.1	4MMs	Chr6:-19265675	LOC_Os06g33090	intron
GGTCAGGCTCAAGTGCTCGGTGG	0.1	4MMs	Chr6:-19271440	LOC_Os06g33100	exon
GGTCAGGCTCAAGTGCTCGGTGG	0.1	4MMs	Chr6:-19258290	LOC_Os06g33080	exon
GGACAGACTCACGTACTCCCAGG	0.1	4MMs	Chr10:+4041974	LOC_Os10g07542	exon

SG640 postion: Chr3:+6090129 Guide sequence: GATGCCGAGGTCCTCGAGCATGG

Predicted off-target sites for SG640 site:

Sequence	Score	MMs	Locus	Gene	Region
GGAGTCGAGGGCCTCGAGCACGG	0.8	4MMs	Chr3:+862609	LOC_Os03g02430	exon
GATGCCGAGGTCCTTGAGCCTGG	0.7	2MMs	Chr4:+1026229	LOC_Os04g02690	exon
GAGGCCGAGGGACTCGAGCACGG	0.7	3MMs	Chr6:-3650122	LOC_Os06g07560	exon
ATTGCCGAGCTCCTCGAGCTCGG	0.7	4MMs	Chr4:+7155034	LOC_Os04g12960	exon
ATTGCCGAGCTCCTCGAGCTCGG	0.7	4MMs	Chr4:+7026047	LOC_Os04g12690	exon
GATGAGGAGGTCGTCGAGCACGG	0.6	3MMs	Chr3:+5590948	LOC_Os03g10880	exon
GATGAGGAGGTCGTCGAGCACGG	0.6	3MMs	Chr5:-26749777	LOC_Os05g46120	exon
GGCGCAGAGGACCTCGAGCACGG	0.5	4MMs	Chr1:+40677374	LOC_Os01g70250	exon
GATGACGAGGTCGTCGAGGACGG	0.4	3MMs	Chr1:+21944968	LOC_Os01g39040	exon

SG641 postion: Chr3:-6089774 Guide sequence: GCAGTGGTCGCTGCACCTGAGGG

Predicted off-target sites for SG641 site:

Sequence	Score	MMs	Locus	Gene	Region
GCGGAGGTCGCTGCACCTCACGG	1	3MMs	Chr8:-20013729	LOC_Os08g32310	exon
GCAGAGGGAGCTGCAGCTGAAGG	0.2	4MMs	Chr2:-31996582	LOC_Os02g52250	exon
GCTGGGGTCGCTGCTCGTGATGG	0.2	4MMs	Chr4:+11721509	LOC_Os04g20880	exon
GAACTGGTCGCTGCAGCTCAAGG	0.1	4MMs	Chr2:+32995893	LOC_Os02g53890	exon
GCCGTGGTCCCTGCATCTGCCGG	0.1	4MMs	Chr6:+597759	LOC_Os06g02054	exon
GGAGTGGTCACTGCAGCTGGCGG	0.1	4MMs	Chr1:+1869038	LOC_Os01g04230	exon
GCGGTGGTCGCAGAGCCTGACGG	0	4MMs	Chr5:-1541161	LOC_Os05g03610	exon

Table S4. The seed-setting rate was reduced in *osppr676* RNAi lines 15 and 19.

Lines	No.of filled grains per plant	No.of total grains per plant	seed-setting rate[a](%)
WT	801.9±18.2	960.7±12.4	83.5±1.4
RNAi line15	358.1±8.1	764.8±13.2	46.9±1.4[b]
RNAi line19	674.1±6.8	995.6±7.5	67.7±0.3[b]

[a]The seed-setting rate was measured based on three replications,each replication has three plants.

[b]Significantly different from control at $p<0.01$ by two-tailed *t*-test.

Table S5.

Total Soluble Fatty Acids of Wild-Type and Mutant Anthers.

Acids	Wild-Type<br>Mean±SD<br>(ug/mg Dry Weight)	*Osppr676-2*	up	*Osppr676-1*	up
C12 acid	0.394 ± 0.055	0.104 ± 0.057	-73.60%	0.090 ± 0.009	-77.16%
C14 acid	0.484 ± 0.171	0.103 ± 0.013	-78.72%	0.372 ± 0.127	-23.14%
C16 acid	7.183 ± 1.223	5.230 ± 0.403	-27.19%	3.564 ± 0.809	-50.38%
C18 (2/3)acid	7.534 ± 0.513	4.273 ± 0.562	-43.28%	3.530 ± 0.120	-53.15%
C18 (0/1)acid	3.190 ± 0.797	2.239 ± 0.065	-29.81%	1.515 ± 0.025	-52.51%
C20(1/2) acid	2.918 ± 0.546	0.715 ± 0.014	-75.50%	0.121 ± 0.049	-95.85%
C20 acid	0.250 ± 0.020	0.183 ± 0.003	-26.80%	0.135 ± 0.004	-46.00%
C22 acid	0.141 ± 0.065	0.069 ± 0.024	-51.06%	0.039 ± 0.006	-72.34%
C24 acid	0.129 ± 0.032	0.054 ± 0.011	-58.14%	0.041 ± 0.008	-68.22%
C26 acid	0.155 ± 0.033	0.086 ± 0.011	-44.52%	0.134 ± 0.053	-13.55%
Total acids	22.377 ± 2.769	13.057 ± 0.171	-41.65%	9.541 ± 0.670	-57.36%
